# Editorial: About the Foodborne Pathogen *Campylobacter*

**DOI:** 10.3389/fmicb.2017.01908

**Published:** 2017-10-10

**Authors:** Odile Tresse, Avelino Alvarez-Ordóñez, Ian F. Connerton

**Affiliations:** ^1^SECALIM, INRA, Oniris, Université Bretagne Loire, Nantes, France; ^2^Department of Food Hygiene and Technology and Institute of Food Science and Technology, University of León, León, Spain; ^3^Division of Food Sciences, School of Biosciences, University of Nottingham, Nottingham, United Kingdom

**Keywords:** foodborne pathogen, epidemiology, host interaction, control strategies, metabolism and regulation, methodology, chicken gut microbiota

## Introduction

The name “Campylobacter” comes from ancient Greek meaning “curved rod” which describes the shape of this microorganism. *Campylobacter* was firstly isolated as a *Vibrio* species from epizootic ovine abortion in 1906 by McFadyean and Stockman (1913), and renamed in 1973 as the neotype strain *Campylobacter* after showing significant biological differences with *Vibrio* species (Véron and Chatelain, 1973). Rather than a curved rod, the shape looks more like to a spiral and can develop in to filamentous or coccoid forms under stressful conditions (Tangwatcharin et al., 2006; Ghaffar et al., 2015; Rodrigues et al., 2016). Nowadays, *Campylobacter* spp. are classified among the ε-proteobacteria in the family of Campylobacteriaceae (Vandamme et al., 1991). *Campylobacter* has emerged as the leading cause of bacterial foodborne infections in developed countries, having surpassed *Salmonella* several years ago, and represents a significant economic burden (EFSA and ECDC, 2016). Although new species of *Campylobacter* have been recently discovered, human cases of campylobacterosis are dominated by two main species, *Campylobacter jejuni* and, to a lesser extent, *Campylobacter coli*.

Quantitative epidemiology reports reveal high rates of contamination for broiler chickens and carcasses by *Campylobacter* (Hue et al., 2010; Lawes et al., 2012; Powell et al., 2012). The presence of *Campylobacter* was also detected in other farm animals or foodstuffs due to cross contamination (EFSA and ECDC, 2016). *Campylobacter* in poultry remains a problem with no effective control measures available that can be recommended for microbial food/farm safety guidelines to mitigate the risk of flock colonization. *Campylobacter* also remains a puzzle as to how an obligate microaerobic bacterium can survive from farm to retail outlets. The underlying molecular mechanisms of persistence, survival and pathogenesis appear to represent a combination peculiar to this pathogen, which are not shared with other foodborne bacterial pathogens such as *Listeria monocytogenes, Salmonella enterica, Escherichia coli*, and *Staphylococcus aureus*.

This topic includes 18 published articles describing original studies of *C. jejuni* and *C. coli* that deal with (1) epidemiology and animal carriage, (2) host interaction, (3) control strategies, (4) metabolism and regulation specificities of these two pathogen species, (5) methodology to improve cultural technique and (6) chicken gut microbiota challenged with *Campylobacter*.

### Epidemiology and animal carriage

Organic animal production schemes differ in many ways (antibiotic use, herd structure, feeding regimes, access to outdoor areas, space allowance) from conventional rearing systems, and therefore can have an impact in the occurrence, transmission and pathogenicity of foodborne pathogens, including *Campylobacter* spp. In this research topic, is however shown that organic pig production schemes have a minor impact on the epidemiology of *C. coli*. Kempf et al. monitored the prevalence and antimicrobial resistance of *C. coli* isolated from conventional and organic pigs in farms from France and Sweden and observed no significant difference in prevalence between pigs in organic and conventional productions. They however observed in France a higher occurrence of antimicrobial resistant *C. coli* isolates (particularly against the antibiotics tetracycline and erythromycin) from conventional rearing systems. Denis et al. characterized *C. coli* isolates obtained from 19 organic pig farms and 24 conventional pig farms through pulsed field gel electrophoresis, multilocus sequence typing, detection of nine virulence-associated genes and evaluation of the adhesion and invasion capacity on Caco-2 cells. They concluded that pig farm management strategies did not influence the diversity and virulence of *C. coli*.

### Host interactions

Ayllón et al. used a proteomic approach to examine relative differences in protein expression levels between *C. jejuni* interacting with human (INT-407) and porcine (IPEC-1) cell lines. The study revealed 366 differentially expressed proteins after 3 h infection and 485 after 24 h. The identities of the protein enabled analysis of the response pathways that indicate differences in the timing of inflammatory responses between the cell lines and comparative down regulation of the signaling pathways that control cell migration, endocytosis and cell cycle progression in the porcine cell line. The authors attribute the differences in the cellular pathway responses to *C. jejuni* exposure as indicative of the processes that establish either infective or commensal behavior respectively in human or porcine hosts.

Upadhyay et al. examined the potential of three phytochemicals generally recognized as safe (GRAS) and applied at sub-inhibitory concentrations to bacterial growth, to prevent or reduce the severity of human infection. Phytochemical treatments of *C. jejuni* resulted in reduced motility and a reduction in the expression of cytolethal distending toxin that could result in favorable effects on human infection. Using human intestinal epithelial (Caco-2) cell-based assays the authors demonstrated the abilities of trans-cinnamaldehyde (0.01%), carvacrol (0.002%), and eugenol (0.01%) to reduce the processes of attachment, invasion and translocation of *C. jejuni*.

### Control strategies

Effective control of *Campylobacter* on commercial broiler chicken farms is proving more than challenging. Microbiological risk assessments suggest that if reductions in the intestinal loads carried by chickens could be translated on to poultry meat then these measures could make a significant impact on the cases of human campylobacterosis (Boysen et al., 2013). Two studies within the topic report the results of feed supplement additions to broiler chicken diets directed to reduce *Campylobacter* colonization levels. Guyard-Nicodème et al. reported the effects of five treatments applied on a French free-range broiler farm that became naturally positive for *C. jejuni* when the birds went on to range after 35 days. A combination treatment of a cation exchange clay-based product and an organic acid mixture containing formic acid, sodium formate, lactic acid and propionic acid resulted in a modest but significant reduction of log_10_ 0.82 CFU per g of cecal contents at day 78 when the birds were scheduled for processing. This treatment was also found to be associated with a significant reduction of log_10_ 0.68 CFU per g on the neck skins of the carcasses of the chickens. Wagle et al. examined the impact of β-resorcylic acid as in-feed supplement and the impact of the phytophenolic on the *in vitro* infection of Caco-2 intestinal epithelial cells. Broiler chickens were challenged with *C. jejuni* at day 7 and fed β-resorcylic acid supplemented feed (0.25 to 1%) until day 14, which resulted in significant reductions up to log_10_ 2.5 CFU per g of cecal contents. Sub-inhibitory concentration also reduced *C. jejuni* motility and their ability to attach and invade Caco-2 cells.

Bacteriophage therapy is a sustainable biological control that has the potential to reduce *Campylobacter* colonization of broiler chickens (Connerton et al., 2011). Lis and Connerton investigated factors that impact on virulent bacteriophage infection of *C. jejuni*. As discussed above motility is a critical trait for successful intestinal colonization and infection by *C. jejuni*. These studies confirmed that motility is also critical for one class of bacteriophage and required for efficient infection by a second. For *C. jejuni* any loss in motility to escape phage infection would result in an inability to colonize animal hosts. As a response to this Achilles heel, campylobacters utilize a second minor flagellin protein, FlaB, to enable regrowth post phage infection. These populations arise still largely at the expense of motility but have the potential to revert to full motility and survive once separated from the bacteriophage. Although, once motility has been recovered, they will become susceptible once more to bacteriophage infection.

### Metabolism and regulation

#### Glucose metabolism

*C. jejuni* is an asaccharolytic micro-organism characterized by the absence of a functional Embden-Meyerhof-Parnas glycolysis pathway due to the absence of glucokinase (Glk) and phosphofructokinase (Pfk). Gluconeogenesis fueled by amino acids is the main pathway for *C. jejuni* to synthesize anabolic hexose phosphate. The Entner-Doudoroff (ED) pathway is an alternative in bacteria to synthesize pyruvate from extracellular glucose via phosphogluconate in order to bypass the absence of Pfk. In this research topic, the key genes of the ED pathway have been found in the genomes of rare isolates of both *C. jejuni* and *C. coli* (Vegge et al.). A complete gene set encoding a functional ED pathway in these *C. jejuni* and *C. coli* isolates are located on a transferable genomic island, similar to that for the genes involved in the utilization of L-fucose in human gut by few *C. jejuni* (Stahl et al., 2011). Interestingly, the presence of a functional ED pathway gives advantages to these bacterial isolates for survival and biofilm formation (Vegge et al.).

#### Oxidative stress response

*Campylobacter* species are obligate microaerobic microorganisms characterized by the inability to multiply in ambient levels of oxygen (Macé et al., 2015). However, some strains are able to better counteract aerobic conditions for their survival. Recently, the atypical *C. jejuni* Bf was described to be able to grow under aerobic conditions in contrast to other *C. jejuni* (Rodrigues et al., 2015, 2016). As for *C. jejuni* Bf, *C. coli* OR12 is also able to develop an habituation to aerobic conditions. In this research topic, the hyperaerotolerance of *C. coli* was found to be associated with an increased resistance to peroxide stress (O'Kane and Connerton). On the other hand, *C. jejuni* is not able to grow anaerobically, even though it can produce functional alternative electron acceptors to O_2_, such as fumarate, nitrate, nitrite, trimethylamine-N-oxide or dimethylsulfoxide (Weingarten et al., 2009). This was attributed to the absence of an alternative to the O_2_-dependent Class I type of ribonucleotide reductase, an enzyme essential for DNA synthesis (Sellars et al., 2002). In this research topic, the regulation and function of various C4-dicarboxylate components as alternative electron acceptors or transporters in *C. jejuni* were explored. It was suggested that the DctA transporter participates in the uptake of succinate at high oxygen levels while *dcuA* and *dcuB* genes, controlled by RacRS system, are up-regulated in oxygen-limited conditions (Wösten et al.). The main enzymes constituting the sub-system of oxygen detoxification in *C. jejuni* has been described. Nonetheless, the regulation of these enzymes remain elusive as the main regulators previously described to play this role in other Gram-negative bacteria (SoxRS and OxyR regulons) are absent in *C. jejuni* genome. These enzymes could be potentially controlled by the iron homeostasis and transcriptional regulating mechanisms including the essential pleiotropic regulator CosR and the inessential RrpA and RrpB regulators. In this research topic, Gundogdu et al. have brought new insights concerning the roles of RrpA and RrpB in the response to oxidative and aerobic stress conditions. The gene *rrpA* is present in over 99% of *C. jejuni* strains while *rrpB* seems to be restricted to livestock clonal complexes. This latter gene is located to a transferable hypervariable region in association with the type I R-M (*hsd*) system. Consequently, the presence of RrpB suggests a specific adaptation of *C. jejuni* to host.

#### Phase transition

Phase transition in bacteria is important for survival and adaptation to harmful conditions. As mentioned earlier, *C. jejuni* is able to modulate its shape in response to environmental conditions. The life cycle of *Campylobacter* is an alternation of states from dormancy to multiplication. Bacterial growth is also punctuated by different phases. The lag phase represents the time for the bacteria to adapt to new conditions, the log phase is characterized by the cell doubling and the stationary phase is a way of survival in growth limiting conditions. The timing of the latter phase is conditioned by the adaptation capability of the cells. It is usually driven by growth factors in response to general stress. In contrast to most of Gram-negative bacteria, the main transcriptional factor, RpoS, which controls the switch between the log and the stationary phases, is absent in *Campylobacter*. Reported in this research topic is the identification of proteins showing significantly different abundances between the two growth phases (Turonova et al.). These proteins belong to biological pathways including metabolism, general and specific oxidative stress response, translation and motility. In addition, the regulator CosR was identified among these differently abundant proteins. The dynamics of the transcript levels of CosR throughout the growth of *C. jejuni* reveal transient differences between the log and stationary phases, suggesting the transcriptional regulator is under negative control. As aforementioned, CosR was originally ascribed a role in the control of enzymes involved in oxygen detoxification. Further analyses indicated that CosR is able to bind to its own promoter region indicating its potential for auto-regulation. The DNA binding consensus sequence of CosR was refined by bioinformatic analysis of the promotor region of CosR and other genes previously described to be able to bind this protein. Although the complete regulatory framework associated with CosR remains to be discovered, these data suggest a major contribution of CosR during the switch between exponential and stationary phases in *C. jejuni*.

#### Characterization of new genes

Whole genome sequencing provides comprehensive set of features by which *Campylobacter* demonstrates genetic variation and plasticity. Many well described molecular mechanisms in bacteria are not transferable to *Campylobacter* due to the absence of homologous genes. Consequently, the mutational analysis of specific genes with putative functions continues to turn up new aspects of the biology of *Campylobacter*. For instance, the putative transcriptional regulator Cj0440c, belonging to the TENA/THI-4 family of proteins, could play a role in compensating the fitness cost of erythromycin resistance through a positive relationship with flagellar proteins (Hao et al.). The protein Cj1199 seems to be involved in the leucine biosynthesis and transport but could also indirectly affect the development of erythromycin resistance in *C. jejuni* (Hao et al.). In another study also published in this research topic (Taylor et al.), the analysis of two putative chaperone genes (Cj1289 and Cj0694) was investigated using a mutational approach. The protein Cj0694 is predicted as an inner membrane anchored protein but possesses a peptidyl-prolyl cis/trans isomerase (PPIase) activity, which could be involved in the initial folding and outer membrane translocation of Cj1289, a SurA-like chaperone (SalC). These two proteins likely participate to the outer membrane protein biogenesis and integrity.

### Methodology to improve cultural technique

Culture-based isolation methods targeting *Campylobacter* spp. are usually challenging for some type of highly contaminated samples, due to its outgrowth by major competing bacteria in the enrichment conditions. In this research topic, an improved culture-dependent methodology for the selective isolation of *C. jejuni* from wastewater samples is described. Kim et al. assessed a few different enrichment conditions using five different antibiotics (i.e., cefoperazone, vancomycin, trimethoprim, polymyxin B, and rifampicin), to which *C. jejuni* is intrinsically resistant. They showed that *Enterococcus* spp. and *Pseudomonas aeruginosa* are major competing bacteria in the enrichment conditions and that the addition of polymyxin B, rifampicin or both to the selective media enhanced the selective isolation of *C. jejuni*.

### Chicken gut microbiota challenged with *Campylobacter*

The gut microbiota plays an essential role in nutrition, feed conversion, growth performance and protection against pathogenic bacteria such as *Campylobacter* spp. However, and despite the increasing number of articles focused on the gut microbiome of humans and animals, there is little information yet about the diversity and function of the gut microbiota in chickens and its impact on the establishment of certain pathogens, including *Campylobacter* spp. This research topic includes a couple of articles dealing with this issue. Thibodeau et al. assessed the impact of feed supplementation with selenium on the gut microbiota of chickens in a *C. jejuni* colonization model. Results obtained by these authors evidenced that, for healthy chickens raised in good hygienic conditions, selenium-yeast did not influence neither the body weight nor the caecal microbiota or the colonization status by *C. jejuni*. Awad et al. monitored in a longitudinal study from day 1 to day 28 of age the composition and structure of the microbiota of the gut content and the mucosa, as well as the consequences of a *C. jejuni* infection on the gut microbiome. These authors show in their article that the chicken gut microbiota significantly changes during the first 28 days of age, that numerous significant differences in microbial profiles are observed between the mucosa and luminal content of the small and large intestine, and that *C. jejuni* colonization is associated with an alteration of the gut microbiota, which confirms that the *Campylobacter* carrier state in chickens is characterized by multiple changes in the intestinal ecology within the host.

## Conclusion

Recent researches indicate that genomic polymorphism, restricted catabolic capacity, self-regulation or deregulation of genes, bacterial cooperation and unknown contamination routes may all be connected to the specificity of pathogenic species of *Campylobacter*.

## Author contributions

OT prepared the content. OT, IC, and AA wrote the paper.

### Conflict of interest statement

The authors declare that the research was conducted in the absence of any commercial or financial relationships that could be construed as a potential conflict of interest.
